# Combining the External Medical Knowledge Graph Embedding to Improve the Performance of Syndrome Differentiation Model

**DOI:** 10.1155/2023/2088698

**Published:** 2023-02-01

**Authors:** Qing Ye, Rui Yang, Chun-lei Cheng, Lin Peng, Yong Lan

**Affiliations:** School of Computer, Jiangxi University of Chinese Medicine, Nanchang, Jiangxi, China

## Abstract

The electronic medical records (EMRs) of traditional Chinese medicine (TCM) include a wealth of TCM knowledge and syndrome diagnosis information, which is crucial for improving the quality of TCM auxiliary decision-making. In practical diagnosis, one disease corresponds to one syndrome, posing considerable hurdles for the informatization of TCM. The purpose of this work was to create an end-to-end TCM diagnostic model, and the knowledge graph (KG) created in this article is used to improve the model's information and realize auxiliary decision-making for TCM disorders. We approached auxiliary decision-making for syndrome differentiation in this article as a multilabel classification task and presented a knowledge-based decision support model for syndrome differentiation (KDSD). Specifically, we created a KG based on TCM features (TCMKG), supplementing the textual representation of medical data with embedded information. Finally, we proposed fusing medical text with KG entity representation (F-MT-KER) to get prediction results using a linear output layer. After obtaining the vector representation of the medical record text using the BERT model, the vector representation of various KG embedded models can provide additional hidden information to a certain extent. Experimental results show that our method improves by 1% (P@1) on the syndrome differentiation auxiliary decision task compared to the baseline model BERT. The usage of EMRs can aid TCM development more efficiently. With the help of entity level representation, character level representation, and model fusion, the multilabel classification method based on the pretraining model and KG can better simulate the TCM syndrome differentiation of the complex cases.

## 1. Introduction

With the fast advancement of the medical information technology in recent years, the number of electronic medical records (EMRs) accessible has increased. EMRs are mostly semistructured or unstructured text records of the diagnostic and treatment processes. EMRs serve as the central repository for big medical data, which contains a wealth of medical information and therapeutic concepts. The course record in medical records can be divided into four diagnostic information, physical examination, chief complaint, syndromes, treatment plan, and traditional Chinese medicine (TCM) prescription.

In general, there are few same syndromes in TCM diagnosis. Usually, a case corresponds to a syndrome, or a patient corresponds to a syndrome. The number of syndromes is far too many to classify. Therefore, we proposed the task of transforming the syndrome differentiation problem into identifying the nature and locations of the disease. This way, we can transform the TCM syndrome differentiation auxiliary decision-making task from the original multiclassification task into a multilabel classification task, where the disease's nature and location are regarded as labels. However, doctors give clinical prescriptions based on the diagnosed syndromes. As a result, extensive TCM clinical expertise and excellent TCM diagnostic understanding are required in the syndrome differentiation approach. In order to imitate the TCM dialectics process, we need to introduce the information implied by the symptoms and chief complaints in each medical record into the medical record text representation. So, we constructed a knowledge graph (KG) based on TCM features (TCMKG) to excavate hidden information in the medical records to solve this problem.

The language model of a deep neural network has developed into an efficient text representation method in recent years. BERT [1] has shown promise in some natural language processing (NLP) tasks. We adopted BERT for EMRs multilabel classification and extended the model with knowledge representation with hidden information provided in the shared task, such as four diagnostic information, chief complaint, and physical examination. Experiments on a test set of EMRs demonstrated the efficacy of our method.

The following are the contributions of this paper: (1) To get around the problem of not being able to use EMRs for information-based dialectics, we turned TCM's standard syndrome differentiation work into a multilabel categorization assignment. (2) In this paper, we proposed syndrome differentiation assistant decision-making based on knowledge (KDSD) to integrate implicit knowledge graph embedding (KGE) from TCMKG into the syndrome differentiation assistant task. (3) We designed a method to adopt the fusion of medical text and knowledge graph entity representation (F-MT-KER) to alleviate the apparent impact of the personalization of the TCM medical records on decision-making.

## 2. Related Works

Machine learning often accomplishes multilabel classification tasks by changing the current algorithm or employing a binary classification technique. Unlike traditional machine learning, the deep learning method uses various neural network structures to extract the semantic embedding of input text. XML-CNN [2] used a one-dimensional convolutional neural network along the sequence length and word embedding dimension to represent the text input. Slice considers supervised pretrained dense embeddings from XML-CNN model as the input of its hierarchical linear model. Recently, based on XML-CNN, AttentionXML [3] used BiLSTM, added attention to labels to design scoring functions, and used a hierarchical tag tree to warm up the model.

Many studies have been committed to pretraining language representation models to obtain language information from a text and then use this information for specific NLP tasks. The Elmo [4], GPT [5], and BERT [1] models have achieved better results in multiple NLP tasks. After fine-tuning, these pretraining models can be applied to various tasks. However, due to the lack of information linkages between vertical applications and open domains, these models cannot be easily translated to specialized domains. The model can be pretrained in specific fields to solve this problem, but it is not desirable in terms of computational time and cost. For example, RoBERTa [6], XLNET [7], Span-BERT [8], and ERNIE [9] are trained in the abovementioned way. Recent research has demonstrated that in addition to the input, adding other relevant knowledge to the model can improve the model's performance to variable degrees, such as reading comprehension [10], text classification [11], natural language inference [12], knowledge acquisition [13], and question answer [14]. Therefore, we argued that additional knowledge information could effectively benefit the existing pretraining model. Some studies have tried effectively controlling the external KGs through the joint representation learning of words and entities to achieve promising results [15–18]. Chen et al. [19] used BiLSTM to process text and introduced additional knowledge through specific attention mechanisms. Zhang et al. [20] tried to use external KG to enrich embedded information and improve language understanding.

In TCM data mining, Zhou [21] built a data model from multiple information entities and their interactions in outpatient data to fulfill large-scale clinical data integration and preprocessing activities, as well as to uncover useful clinical knowledge from the data. Liu et al. [22] approached a clinical data warehouse based on structured EMR data. Clinical terminology is collected to establish clinical hypotheses and aid in the discovery of clinical knowledge from large-scalereal-world TCM clinical data. The majority of TCM data mining involves constructing data into a database and then mining information from it to realize the application [23]. In recent years, the auxiliary decision-making systems based on expert systems [24] and the model-assisteddecision-making systems [25] based on deep learning have dominated TCM diagnosis. Joyce et al. [26] described the cognitive work of TCM tongue diagnosis and used it to find TCM diagnostic thinking patterns. An expert panel must assess the correctness of the diagnosis. Xie et al. [27] mined links between hidden items and inferred pathways from symptoms to syndromes using TCM knowledge graphs and reinforcement learning techniques. Yang et al. [28] utilized KGE to find triples connection pairings of syndromes and symptoms, and then created a score algorithm to find suggested syndromes. To apply clinically aided diagnostic and treatment models, Ruan et al. [29] stacks semantic-aware graph convolutions to learn efficient low-dimensional representations of nodes via meta-graphs and self-attention, and predicts correct patterns via clustering and linking. Xie [30] employed multiclassifier integrated TCM syndrome element classification to help with diagnosis, mainly using the Nave Bayes, Weighted bipartite graph, SVM, and ProSVM algorithms. Since the number of syndromes in actual diagnosis is unclear, we split syndromes into labels of illness type and disease location, used multilabel classification to predict labels, and utilize a certain number of labels to replace ambiguity.

## 3. Methods

### 3.1. Overview

**Figure 1:**
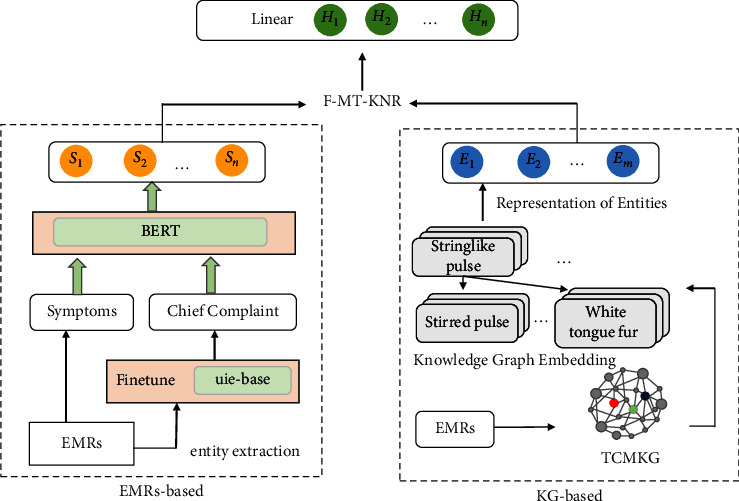
The architecture of the KDSD model.

The KDSD model is divided into two components, as illustrated in Figure 1, the EMRs module and the KG module. We first develop the TCMKG using the EMRs and then represent all entities and relationships in KG through the KG embedding model. Input the EMRs into the model, and the EMRs module will extract the chief complaint and symptom information (four diagnostic information and physical examination). Meanwhile, the embedding representation of the knowledge entities in the KG module is obtained by extracting and linking the entities of the EMRs module. Finally, we fused text representations and KG entity embedding representations through the F-MT-KNR method designed in this paper to predict the nature and location of diseases through multilabel classification. The implementation details of the model are described below.

### 3.2. Construction of Knowledge Graph

Different doctors use different syndrome differentiation procedures, which results in them prescribing different syndromes for the same illness and even achieving different syndrome outcomes for the same patient. In TCM, syndromes include the relationship between evil and positive, internal and external pathogenic factors, the location of the disease, and the nature of the disease. Therefore, it is objective to use the nature and location of the disease to assist doctors in making clinical decisions.

**Figure 2:**
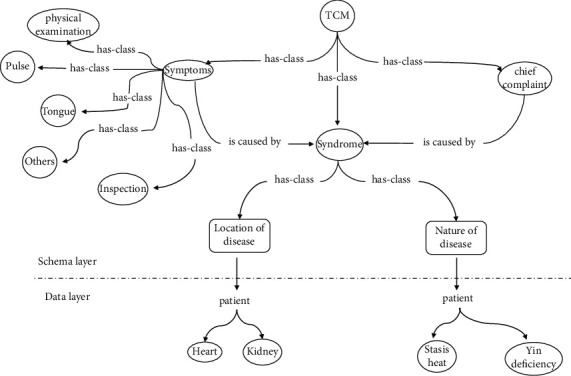
Ontology representation of TCMKG.

In the data mining phase, we extracted the TCM symptom entities and chief complaint information from the desensitized EMRs. We then decomposed the information on the nature of the illness and its location in the syndrome. We designed the schema layer top-down [31], which is actually to classify these entities. As a result, the schema layer's categories and the data layer's medical entities establish “class-example” connections. The data layer is filled with entities simultaneously, and the TCMKG is completed.  Figure 2 shows the partial TCMKG.

After desensitization, entity extraction, and relationship extraction from 12533 EMRs, we created TCMKG. The TCMKG database has 115012 triples, which include 18078 symptom entities, 2098 chief complaint entities, and 157 nature and location of the disease entities. In addition, Neo4j is employed as a data storage tool for TCMKG.

### 3.3. Representation of Knowledge Graph

In the EMRs of TCM, there is a very complex relationship between the four diagnostic information and syndrome or disease nature and location, which experts of TCM can only understand. By contrast, KG is a multirelational graph comprised of many different entities and their relationships [32]. In this paper, KG can accurately describe the relationship between symptoms and syndromes in TCM through information extraction, data mining, and knowledge reasoning. It can describe the evolution process and development law of knowledge. To provide accurate, traceable, interpretable, and inferential knowledge data for syndrome auxiliary decision-making. Besides, the representation learning of KG is the method of transforming these knowledge data with implicit information into a vector representation.

Given a knowledge graph Κ, which includes a collection of entities *ℇ* and relations *ℛ*, that is, Κ⊆*ℇ* × *ℛ* × *ℇ*. The triples are expressed as (*h*, *r*, *t*), where *h*, *r* ∈ *ℇ* denote the head and tail entities, respectively, and *r* ∈ *ℛ* denotes the relationship between them.

For each entity *e* and relationship *r* in Κ, the knowledge graph representation methods generate *e*_*v*_ ∈ *R*^*d*_*e*_^ and *r*_*v*_ ∈ *R*^*d*_*r*_^, where *e*_*v*_ and *r*_*v*_ are *d*_*e*_ and *d*_*r*_ dimensional vectors, respectively. Each embedding technique incorporates a scoring mechanism, *ℱ* : *ℇ* × *ℛ* × *ℇ*⟶*R* will give some scores *ℱ*(*h*, *r*, *t*) is assigned to a possible triple (*h*, *r*, *t*), *h*, *t* ∈ *ℇ*,, and *r* ∈ *ℛ*. The model is trained in such a way that for each correct triple (*h*, *r*, *t*) ∈ Κ and wrong triple (*h*′, *r*′, *t*′) ∉ Κ, the model assigns a score such that *ℱ*(*h*, *r*, *t*) > 0 and *ℱ*(*h*′, *r*′, *t*′) < 0. Typically, a scoring function is a function of (*e*_*h*_, *e*_*r*_, *e*_*t*_).

We selected several representative KG embedding methods to conduct experiments on TCMKG (see the experimental part) and evaluated the indicators, among which TransE performed best.

Bordes et al. [33] proposed TransE based on the assumption that the additional *h*+*t* embedding should be near to that of t and that the scoring function should be defined as follows under *L*_1_ or *L*_2_ constraints:

(1) $$\begin{array}{c} f_{r}\left(h,t\right)={\left\|h+r-t\right\|}_{L1/L2}\text{.} \end{array}$$

As mentioned above, *h*, *t* ∈ *ℇ*, and *r* ∈ *ℛ*. After the KG embedding is completed, formula (1) makes *f*(*h*, *r*, *t*) > 0 for all true triples, and *f*(*h*, *r*, *t*) < 0 for all wrong triples.

### 3.4. EMR-Based Module

The BERT layer is used by the EMRs module to extract features from incoming text input. The embeddings of the KDSD model differ from those of the BERT model. We use outside information to complement the text's representation. We separate the information about the principal complaint from the information about the symptoms when we analyze the input data, and the format of the input sequence is specified by

(2) $$\begin{array}{c} \left[CLS\right]symptoms\left[UNK\right]chief\;complaint\left[UNK\right]\left[SEP\right]\text{,} \end{array}$$

where [CLS] and [SEP] are the BERT model-specific special symbols, and [UNK] is the separator inside the text. Symptoms include pulse-taking, tongue examination, listening and smelling, inspection, and physical examination. Each symptom and chief complaint field is separated by [UNK] to facilitate the integration of subsequent KGE.

After entering the EMRs into the model, it is required to improve the text representation via the hidden layer. However, the symptom information and the chief complaint information are stated differently in EMRs, as is the length of the text. Since the maximum input length of BERT is generally 512 characters, considering that this paper needs to separate symptoms and chief complaints, and the description of private complaints is too complicated, the length of some medical records exceeds the limit of the model. In this paper, most of the information on the four diagnostic information is relatively standardized. To decrease the text length, we apply the fine-tuned Uie-base [34] model to extract the main complaint information from the text. Then, as stated in the following equations, we employed the multi-headself-attention [35] process to integrate the principal complaint information into the text representation of symptom information.

(3) $$\begin{array}{c} Q=K=V=W^{S}Concat\left(\left[C\right];S_{1\ldots M},C_{1\ldots N}\right)\text{,} \end{array}$$

(4) $$\begin{array}{c} \left[C^{\prime }\right]=Concat\left({head}_{1},\ldots ,{head}_{i}\right)W^{O}\text{,} \end{array}$$

(5) $$\begin{array}{c} {head}_{i}=Attention\left(QW_{i}^{Q},KW_{i}^{K},VW_{i}^{V}\right)\text{,} \end{array}$$

where [*C*] is the representation of [CLS] in the hidden layer state, [*C*′] is the representation of input text. *S*_1…*M*_ and *C*_1…*N*_ are symptom information embedding containing *M* values and chief complaint information embedding containing *N* values. *W*^*s*^,*W*^*O*^, *W*^*Q*^, *W*^*K*^, and *W*^*V*^ are trainable parameters.

Integration of additional medical knowledge.

**Figure 3:**
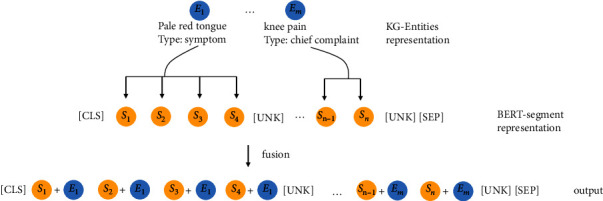
The process of fusion of knowledge graph entity information and character information of the BERT model.

The fusion information part aims to integrate the output of KGE into the output of BERT. BERT is to train the representation of each character in the unit of characters, while in the KGE module, we can only train the entities in KG into triples. Therefore, we proposed F-MT-KER to realize the fusion of characters and entity embeddings in KG, as shown in Figure 3.

(6) $$\begin{array}{c} S=\overset{i=k}{\underset{i=1}{\sum }}\left[\left(S_{j},\ldots ,S_{j+L\left(e_{k}\right)}\right)+e_{k}\right]\text{.} \end{array}$$

In equation (6), *e*_*k*_ is the embedding of an entity in KG, *S*_*j*_,…, *S*_*j*+*L*(*e*_*k*_)_ corresponds to symptom character set or chief complaint character set of *e*_*k*_. L() is the function of calculating the length of the entity and *f* is to select the corresponding *e*_*k*_. The embedded representation of the same entity is added to the characters of each entity. Through the linear layer transformation, the fused vector dimension is changed from the dimension of the hidden layer to the dimension of the label quantity.

## 4. Results

### 4.1. Overview

The process of auxiliary decision-making for syndrome differentiation can be divided into the KG construction and embedding module, the text representation of the BERT model, and multilabel classification based on model fusion. For the medical records of the input model, we first take the medical record entity set through TCMKG and then link the entities in this entity set to the vector space embedded in TCMKG through entity matching. As a result, we can obtain vector representations of symptom entities and chief complaint entities. Ultimately, the nature and location of the disease recommended by the medical record can be obtained by inputting the embeddings containing hidden information into the model.

### 4.2. Dataset and Experimental Setup

**Figure 4:**
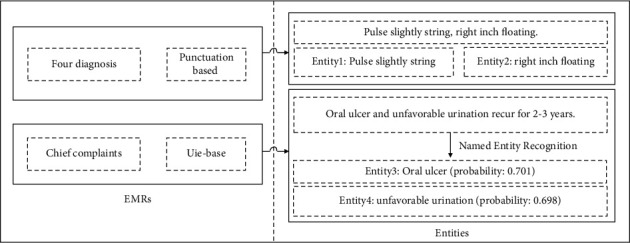
Entity extraction method for four diagnosis and chief complaint information.

We conducted experiments on the EMRs dataset and TCMKG. As seen in Figure 4, we removed sensitive information from the original EMRs and extract entities for pulse diagnosis, tongue diagnosis, and the other four diagnostic information based on the punctuation marks. After fine-tuning the medical record annotation data, the symptom information in the chief complaint is retrieved using the Uie-base model. For example, from the text information, the symptoms of “oral ulcer” and “unfavorable urination” may be extracted from “oral ulcer and unfavorable urination” that recur repeatedly for 2-3 years. It is worth mentioning that when the probability of the anticipated symptom item is less than 0.5, the entity is deleted.

The dataset includes 12533 EMRs from QIHUANG TCM. 10026 EMRs were used for training and 2507 were used for testing. Furthermore, our criterion for dividing the test set is that only the test data labels that appear in the training set can be divided into the test set. This part of the data accounts for a tiny proportion, so we used the model to generate their input randomly. There are 157 classifications for disease nature and disease location. Every text in the input model is a medical case, and each text has an average of 102 characters. The relationship, number of edges, and triples in the knowledge graph constructed in this paper are shown in Table 1.

We constructed the knowledge ontology based on Yu [31] and defined entity and relationship description as the core. It includes some knowledge in EMRs, such as pulse diagnosis, tongue diagnosis, smell diagnosis, observation diagnosis, chief complaint, and other knowledge.

This paper preprocessed EMRs through data desensitization, cleaning, structuring, filtering, and diagnostic label standardization. In data filtering, duplicate information, and information that has little impact on diagnosis (such as the date in chief complaint) will be deleted. On the one hand, it can fulfill the BERT model input's format criteria. In TCMKG, on the other hand, it may also maintain concealed information. Our BERT model was bert-base-chinese, with the following primary settings: hidden size 768, maximum position embedding 512, number of epochs 50, number of attention heads 12, number of hidden layers 12, maximum input length 256, learning rate 2*e* − 5, and batch size 8. Our NVIDIA T4 GPU is the backbone of all our experiments (17.18G).

### 4.3. Evaluation Metrics

Table 2 displays the experimental outcomes on the EMRs dataset. P@k (Precision at k) was used as evaluation metrics.

(7) $$\begin{array}{c} P@k=\frac{1}{k}\overset{k}{\underset{l=1}{\sum }}y_{rank\left(l\right)}\text{.} \end{array}$$

The index of the *l*th highest prediction label is rank(*l*) and *y* is the true binary vector.

#### 4.3.1. Hamming Loss

The Hamming loss is the proportion of wrongly predicted labels. The higher the performance, the lower the Hamming loss value. $\hat{y_{i}}$ is the predicted value for the *j*th label of a given sample, *y*_*i*_ is the corresponding true value, and *n*_labels_ is the number of classes or labels.

(8) $$\begin{array}{c} L_{Hamming}\left(y,\hat{y}\right)=\frac{1}{n_{labels}}\overset{n_{labels}-1}{\underset{j=0}{\sum }}1\left(\hat{y_{i}}\neq y_{i}\right)\text{.} \end{array}$$

#### 4.3.2. Average Precision

The weighted mean of precisions acquired at each threshold is used to summarize a precision-recall curve, with the increase in recall from the previous threshold used as the weight. Where *P*_*n*_ and *R*_*n*_ are the precision and recall at the *n*th threshold.

(9) $$\begin{array}{c} AP=\underset{n}{\sum }\left(R_{n}-R_{n-1}\right)P_{n}\text{.} \end{array}$$

#### 4.3.3. Label Ranking Loss

The label ranking loss function computes the ranking loss, which is weighted by the inverse of the number of ordered pairs of false and true labels.

(10) $$\begin{array}{c} {ranking}_{loss\left(y,\hat{f}\right)}=\frac{1}{n_{samples}}\overset{n_{sample-1}}{\underset{i=0}{\sum }}\frac{1}{{\left|\left|y_{i}\right|\right|}_{0}\left(n_{labels}-{\left|\left|y_{i}\right|\right|}_{0}\right)}|\left\{\left(k,l\right):\hat{f_{ik}}<\hat{f_{il}},y_{ik}=1,y_{il}=0\right\}\text{.} \end{array}$$

### 4.4. Performance on EMRs Dataset

Liu et al. [36] used a data improvement and model fusion technique to accomplish syndrome differentiation classification of lung cancer diagnosis using the neural network text classification models TextCNN [37], TextRNN [38], RCNN [39], and fastText [40]. Hu et al. [41] employed the CNN and fastText models to complete the task of yin and yang deficient syndrome text categorization.

In addition, this study employs AttentiveConvNet [42], DPCNN [43], Transformer [35], and AttentionXML as comparative models for the multilabel text classification problem. A variety of context information is added to local convolution processes by AttentiveConvNet. Long-distance text dependencies may be extracted using the DPCNN. AttentionXML is a structural model for tag trees based on the attention mechanism. Using a multilabel attention approach for text multilabel categorization, the most essential feature information is collected for each label, allowing the relationship information between the input text and each label to be properly comprehended. Moreover, the KG-based algorithm used the KG's information to forecast relationships that will deliver the desired results. This paper proposes the KDSD model. Bert-base-chinese is the pretraining model that is employed. By linking the linear layer after feature extraction using the model, a multilabel classification result is achieved.

### 4.5. The Results of Knowledge Graph Representation

In order to demonstrate the KGE model's efficacy, we used the mainstream KGE methods [33, 44–46] to evaluate the TCMKG. For each KGE approach, we produce the associated knowledge representation and add it to the KDSD model.  Table 3 illustrates the KDSD model's performance when various KGE models are employed as knowledge supplements.

We employ the MRR (Mean Radical Ranking), MR (Mean Rank), and Hits@N to analyze the performance metrics of KGE [47]. The MRR is the average of the triples' reciprocal rankings. The value ranges from 0 to 1; the greater the number, the better the model. MR is the average of all the triples' rankings. The value ranges from 1 (ideal condition, all rankings are equal) to the number of corruptions. Hits@n is the proportion of calculated ranks that are higher than (or equal to) a rank of *n*. The value ranges from 0 to 1; the greater the number, the better the model.

## 5. Discussion

### 5.1. Principal Results

As seen in Table 2, based on pretraining models, BERT and KDSD models outperform fastText, TextCNN, Text RNN, Transformer, and other models. KDSD increases by 1%, 0.3%, and 0.52% in the metrics P@1, P@3, and P@5, respectively, as compared to the BERT model.

From the experimental results, the results of the pretraining model are better than the previous text-based multilabel classification method. Furthermore, including KGE information in the pretraining model can improve the model's performance. However, all KG-Based indicators were terrible, far lower than the KDSD results. This might be due to one of the two factors. One is that KG-based method only used the information embedded in the graph and obtains the labels through link prediction. Another possibility is that TCMKG is a KG constructed for TCM diagnosis. Although the diagnosis of each medical record is different, the reuse rate of the information of the four diagnoses and the chief complaint is high. Besides, various clinicians define diseases differently, the number of triples for some conditions may be inadequate for good prediction.

From Table 3, it can be seen that the average precision of the KDSD model is 0.9% higher than that of the BERT model. This is due to the fact that in actual text task processing, some training corpora are difficult to obtain; their overall number and the total number of words contained are very small, making them unsuitable for training models with embedding layers. However, these data are suitable for training models without embedding layers. In addition, it provides useful rules that may be extracted by the model. Using a pretrained model to encode the original text is an excellent choice in this instance because the pre-trained model is derived from a large corpus and can make the current text meaningful, despite the fact that these implications may not be domain-specific. However, these deficiencies can be remedied by utilizing fine-tuned models.

### 5.2. Results of KGE

The KG-based experiment is another experiment undertaken for this investigation. If only the knowledge graph and its embedded data are used, the link prediction is performed on multiple triples associated with the same medical record, followed by the application of the KG's embedded scoring function. The expected labels received were evaluated, and the label with the highest score was finally selected. The results are bad due to the high rate of recurrence of the four diagnoses in TCM, the excessive number of triple diagnoses that may be derived from a medical file, and the inability to establish a connection between them. When this experiment is compared to the KDSD model and the BERT model, it is clear that using the information of the KG alone will not provide excellent results; instead, a deep learning model must be used in conjunction with it to get superior outcomes.

Although the KG-based method has no advantages in various indicators, the results of KDSD are indeed better than BERT in some indicators. It shows that the fusion with the embedded information from TCMKG can improve the performance of syndrome decision-making model. Moreover, as shown in Table 3, KDSD has the best performance after using the TransE model, surpassing BERT in all indicators. However, the results of some models have even declined after being fused with BERT. Here we evaluated the performance of KGE.  Table 4 shows the performance evaluation findings for several embedding strategies. As seen in the table, TransE outperforms other models in MRR, MR, Hits@N, and has the greatest performance in the KDSD model for link prediction.

We analyze the reasons for the following reasons. First, complicated embedding approaches, such as adding complex vector space and convolution methods, will not function when the repeat rate of the four diagnosis information and the principal complaint information is high. Second, since the triples of TCMKG construction adopt “symptom-nature\location” and “chief complaint-nature\location,” we think the TransE model's distance-based scoring function calculates the Euclidean distance between them, which has more extraordinary expression ability, utilizing this intuitive distance-based technique.

### 5.3. Limitations

In this section, we discuss some limitations of the KDSD model. First, the new medical record cannot ensure that the input symptoms and chief complaints can match the entities in the TCMKG to ensure the existing label. Therefore, the embedded knowledge integrated into the model has little information, affecting the model's performance. Second, due to the particularity of TCM dialectics, although there is enough information on the syndrome, some syndromes' descriptions are still too simple. For example, if the syndrome is damp-heat obstructing the meridian (湿热阻滞经脉), then, we can get that the location of the disease is the meridians (经脉), and the nature of the disease is obstruction (阻滞) and damp-heat (湿热). This syndrome contains little information about the nature and location of the disease, so such medical records will have an adverse impact on the model's prediction of multiple labels when tested.

## 6. Conclusion

The auxiliary syndrome differentiation work has been viewed as a multiclassification issue. This paper presents the KDSD model for converting multiclassification into a multilabel classification. The KDSD model improves the model's performance by integrating the textual information of the EMR and the entity information embedded in the KG. We constructed TCMKG by using EMRs. Then, we represented TCMKG by TransE, and connected the results to the input layer of BERT. Finally, the results are obtained through the full connection output of the model. The results show that although BERT has been an excellent multilabel classification model in recent years, adding domain-specific knowledge graph information can improve the model's performance.

In the future, we will further study the construction of KG in TCM and explore the critical technologies of model fusion to improve the accuracy of the syndrome differentiation decision-making system. We find that some symptom entities in EMRs are not included in TCMKG (we only consider the existence of labels when building the dataset, some symptom entities will generate vectors randomly when predicting) to introduce other TCM knowledge that contains more symptom, and syndrome entities is an effective feature for syndrome differentiation.

## Figures and Tables

**Table 1:** Number of relation categories and examples of triples in TCMKG.

Relationship categories	Number of edges	*Triples*	
		Chinese	English
Chief complaint-syndrome	16277	<失眠, 主诉_证候, 胆热痰扰>	< Insomnia, chief complaint-syndrome, gallbladder heat, and phlegm disturbance>
Symptom-syndrome	62308	<两寸略弱, 主诉_证候, 胆热痰扰>	< Two inches slightly weaker, symptom-syndrome, gallbladder heat, and phlegm disturbance>
Syndrome-nature of disease	19327	<胆热痰扰,证候_病性, 热>	< Gallbladder heat and phlegm disturbance, syndrome-nature of disease, and heat>
Syndrome-location of disease	17100	<胆热痰扰,证候_病位, 胆>	< Gallbladder heat and phlegm disturbance, syndrome-location of disease, and gallbladder>

**Table 2:** The results on the EMRs dataset.

Models	P@1	P@3	P@5	Average precision	Hamming loss	Label ranking loss
AttentiveConvNet	0.416	0.404	0.384	0.263	0.081	0.136
DPCNN	0.690	0.63	0.574	0.528	0.045	0.079
Transformer	0.721	0.665	0.61	0.578	0.04	0.086
AttentionXML	0.749	0.702	0.645	0.537	0.043	0.078
TextCNN	0.753	0.686	0.636	0.603	0.039	0.069
TextRNN	0.769	0.719	0.668	0.646	0.034	0.068
FastText	0.809	0.748	0.686	0.676	0.034	0.046
BERT	0.89	0.876	0.845	0.857	0.026	0.016
KG-based	0.059	0.073	0.066	—	—	—
KDSD	0.9	0.879	0.852	0.866	0.029	0.014

**Table 3:** Performance of different embedding models on KDSD.

Methods	P@1	P@3	P@5
BERT	0.89	0.876	0.845
+TransE	0.9	0.879	0.852
+DistMult	0.878	0.856	0.829
+ComplEx	0.868	0.852	0.825
+ConvKB	0.886	0.862	0.833

**Table 4:** Performance evaluation of knowledge graph embedding.

Methods	MRR	MR	Hit@1	Hit@10	Hit@100
TransE	0.827	171	0.764	0.912	0.946
DistMult	0.203	492	0.13	0.357	0.633
ComplEx	0.201	530	0.128	0.35	0.625
HolE	0.208	1103	0.133	0.363	0.539
ConvKB	0.157	1011	0.072	0.361	0.551

## Data Availability

The data used to support the findings of this study are available from the corresponding author upon request.

## Abbreviations

EMRs: Electronic medical records
TCM: Traditional Chinese medicine
KG: Knowledge graph
KDSD: Knowledge-based decision support model for syndrome differentiation
TCMKG: Knowledge graph based on TCM features
F-MT-KER: Fusing medical text with KG entity representation
KGE: Knowledge graph embedding.
